# High-grade Pleomorphic Sarcoma Associated with Metallosis in a Patient with Total Hip Arthroplasty

**DOI:** 10.1055/s-0041-1735140

**Published:** 2021-10-13

**Authors:** Roni Serra Campos, Walter Meohas, Artur Shioji Ferradosa, Anneliese Fortuna de Azevedo Freire da Costa, João Antonio Matheus Guimarães, Maria Eugenia Leite Duarte

**Affiliations:** 1 Centro de Cirurgia do Quadril, Instituto Nacional de Traumatologia e Ortopedia, Rio de Janeiro, RJ, Brasil; 2 Centro de Ortopedia Oncológica, Instituto Nacional de Traumatologia e Ortopedia, Rio de Janeiro, RJ, Brasil; 3 Divisão de Pesquisa do Instituto Nacional de Traumatologia e Ortopedia, Rio de Janeiro, RJ, Brasil

**Keywords:** arthroplasty, replacement, hip, hip prosthesis, metals, sarcoma

## Abstract

Although the relationship between hip arthroplasty and the development of sarcoma was first described in the literature about forty years ago, this association is extremely rare. In the present case report, we describe the association between orthopedic implants and soft tissue sarcoma in a 79-year-old man who underwent primary total hip arthroplasty (THA) for coxarthrosis 24 years ago. In the present case report, we describe the clinical evolution and the radiographic and histopathological findings of the lesion. In the intraoperative period of the second revision surgery, loosening of the acetabular and femoral components in association with extensive areas of necrosis and metallosis was evidenced. We performed debridement of the hip and right thigh region and removed the implants. Due to the extent of the lesion and to necrosis, it was not possible to perform a new joint reconstruction. The histopathological diagnosis of high-grade undifferentiated pleomorphic sarcoma associated with extensive areas of metallosis was confirmed in tissue adjacent to the implant. The patient developed pulmonary metastases and died 6 months after the diagnosis. Despite the rarity of this association, sarcomas should be considered in the differential diagnosis of aseptic loosening, especially in the presence of metallosis in the peri-implant tissue. To our knowledge, the 24-year latency period between primary THA and the establishment of a sarcoma diagnosis is one of the longest reported to date.

## Introduction

The cellular response to debris particles resulting from the degradation of different types of materials constitutes the biological basis of peri-implant osteolysis that results in failures in total hip arthroplasties (THA). 1 Wear debris from the components of the prosthesis activate the monocyte-macrophage system, promoting the release of proinflammatory cytokines, in particular growth factors, interleukins, and the receptor activator of nuclear factor-κB ligand (RANKL). The integrated action of these cell signaling molecules, and in particular the activation of osteoclastogenesis through the interaction of RANKL with its receptor activator of nuclear factor-κB (RANK), begins the osteolysis process. Activation of the RANK/RANKL system results in increased recruitment and activity of osteoclasts at the interface with the implant, leading to bone resorption, loosening, and prosthesis failure. 1

The relationship between hip arthroplasty and the development of sarcoma was first reported in 1984. 2 In a review covering a period of 30 years (1974–2003), the occurrence of 46 malignant tumors (41 sarcomas, 4 lymphomas, and 1 squamous cell carcinoma) related to THA was described. 3

## Case Report

**Fig. 1:**
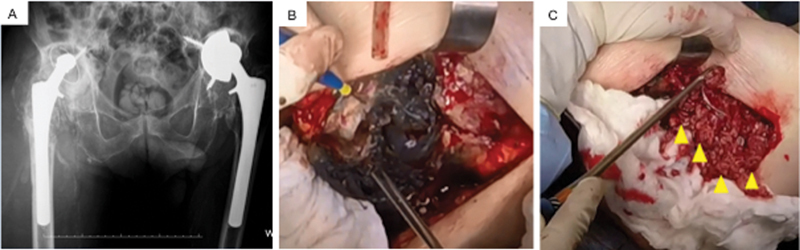
Right hip revision surgery in May 2016. ( **A** ) Anteroposterior panoramic radiograph of the pelvis showing bilateral total hip arthroplasty. On the right, there are signs of resorption of the proximal femur and migration of the acetabular component into the iliac bone, which presents diffuse osteolysis. There is also an important increase in soft tissue volume in the periprosthetic region. The left uncemented prosthesis presents loosening of the acetabular component, which is medialized and verticalized. The femoral component is fixed. ( **B** ) Intraoperative images showing metallosis (blackened areas). ( **C** ) Extensive areas of tissue necrosis adjacent to the implant (arrows) that were debrided to remove the acetabular and femoral components, making it impossible to perform a new joint reconstruction.

**Fig. 2:**
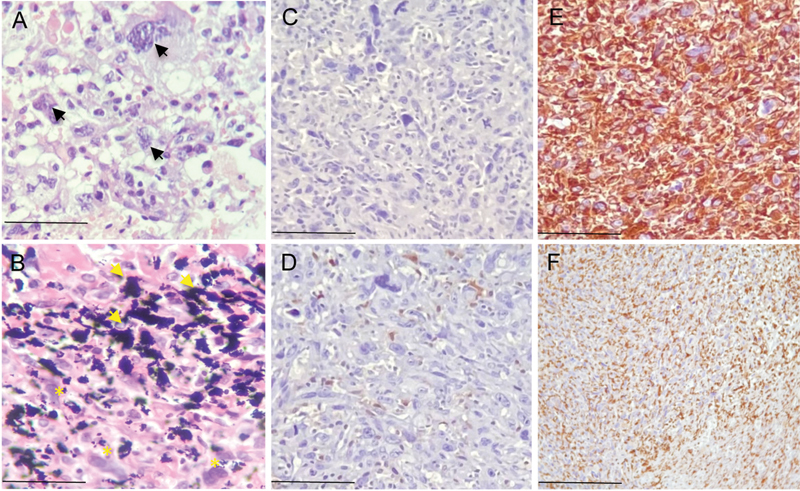
Histopathological aspect and immunohistochemical profile of the lesion associated with the implant in the right hip joint. ( **A** ) High-grade undifferentiated pleomorphic sarcoma characterized by a marked degree of cell anaplasia (arrows). ( **B** ) Wear debris of prosthetic components identified by blackened pigments (arrows) in association with tumor cells (*). ( **C** ) Absence of immunostaining for desmin. ( **D** ) Weak immunopositivity for S-100 protein. ( **E** ) Diffuse positive expression of anti-CD68 antibodies and ( **F** ) Anti-Vimentin. ( **A** and **B** ) Hematoxylin and Eosin (H&E) staining. Scale bar: 50 µm ( **A, B** ), 70 µm ( **C-E** ) and 100 µm ( **F** ).

**Fig. 3:**
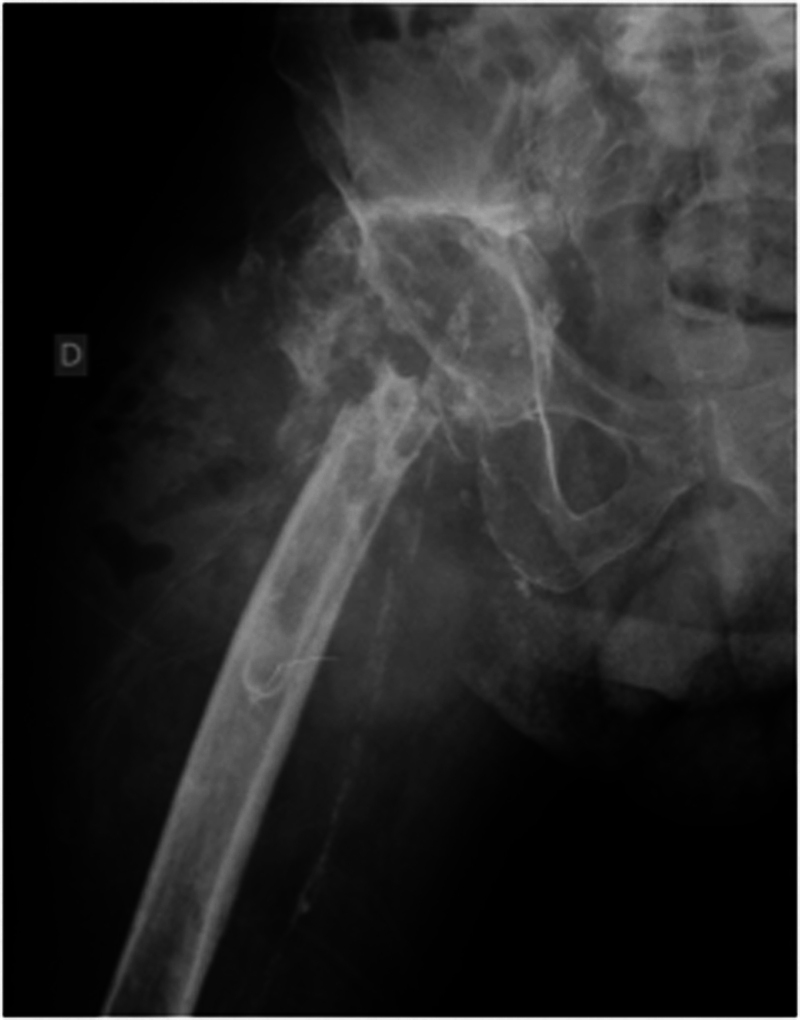
Right hip radiograph performed in July 2016 after revision arthroplasty procedure through posterolateral access with the removal of prosthetic components. Presence of a large tumoral mass in soft tissues in association with extensive destruction of the proximal third of the femur.

**Fig. 4:**
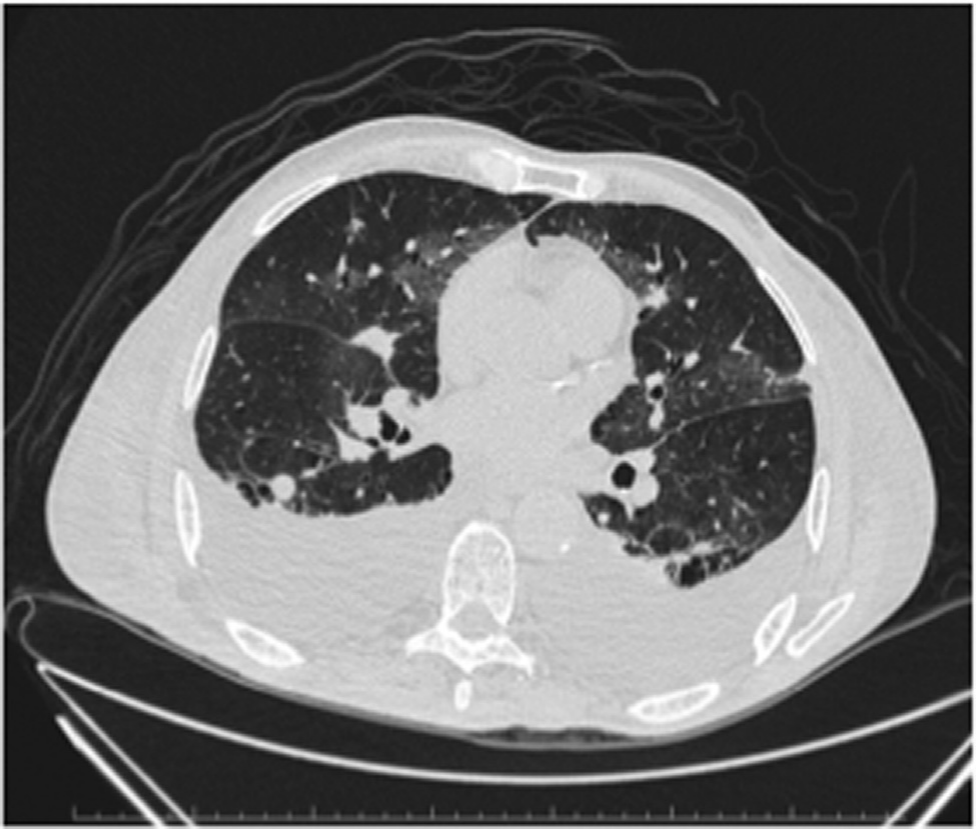
Chest computed tomography scan. In July 2016, the patient had pleural effusion and multiple metastatic nodules in both lungs and died in August 2016.

A 79-year-old white male with bilateral idiopathic coxarthrosis underwent primary THA in 1992 (right hip) and 1993 (left hip). In both surgeries, an uncemented acetabular component was used, made of titanium alloy coated with porous plasma, fixed with acetabular screws, polyethylene acetabular core, and uncemented femoral nail, made of titanium alloy and rough proximal surface, covered with hydroxyapatite and interchangeable cobalt chrome head. In January 2001, the patient presented with loosening of the acetabular component of the left hip and underwent a revision arthroplasty changing for an uncemented acetabular prosthesis. In September of the same year, due to the same diagnosis, he underwent another revision arthroplasty of the right hip, with maintenance of the nail and replacement of the acetabulum for a cemented component. During both surgical procedures, significant wear of the polyethylene and extensive foci of metallosis in the peri-implant tissue were observed. In February 2016, he presented with increased volume and severe pain in the right hip and gait difficulties. On physical examination, he presented with increased thigh volume without fluctuation, decreased range of motion, and was unable to bear weight on the limb, without neurological deficit. Two months after the onset of symptoms, a new revision procedure was performed on the right hip; infection was the main diagnostic hypothesis, and the differential diagnosis was pseudotumoral lesion caused by metal debris. Intraoperatively, the loosening of the acetabular and femoral components and extensive areas of tissue necrosis and metal debris affecting the entire periarticular region was observed, with no evidence of purulent discharge ( Fig. 1 ). The debridement of the hip and right thigh region was performed to remove the implants and to collect material for microbiological culture and histopathological evaluation. Due to the extent of tissue necrosis, it was not possible to perform a new joint reconstruction. The histopathological evaluation confirmed the diagnosis of high-grade undifferentiated pleomorphic sarcoma (UPS) and metallosis ( Fig. 2 ). The results of intraoperative microbiological cultures were negative. In July 2016, the radiography of the right hip showed a large tumoral mass in soft tissues and extensive destruction of the proximal third of the femur. ( Fig. 3 ). The patient evolved with weight loss and dyspnea, being diagnosed with pleural effusion and multiple metastatic nodules in the lungs ( Fig. 4 ), and died in August 2016, 24 years after primary THA.

## Discussion

The incidences of soft tissue sarcomas in the general population and in association with THA were estimated at between 0.5 and 2.0 per 100,000 individuals per year 4 and at 1.43 per 100,000 individuals per year, 3 respectively. The most frequent association with implants is high-grade UPS (malignant fibrous histiocytoma, according to the old nomenclature). However, the association between THA and other sarcomas such as osteosarcoma, angiosarcoma, and synovial sarcoma has also been described. 5 6 7 In a large population study, it was shown that metal-to-metal hip implants are not associated with an overall increased risk of cancer or of cancer deaths over a median follow-up period of 7.4 years. 8 However, this study did not include implants of the same type used in the present report (metal-on-polyethylene).

Considering the date of onset of the complaints of the patient that were compatible with neoplastic disease (local tumor, weight loss, and dyspnea) with the histological confirmation of the diagnosis of UPS, the interval between the primary THA and, therefore, the development of neoplasm was 24 years. This timeframe is considerably longer than the average time of 6.7 years 3 for the development of soft tissue sarcomas associated with orthopedic implants.

Carcinogenesis induced by wear particles and metal corrosion was investigated *in vitro* by exposing fibroblasts to cobalt and chromium particles. The metal particles promoted the generation of reactive oxygen species that resulted in aneuplody, chromosome alterations, mitochondria fragmentation, and damage to the microtubule network that forms the cytoskeleton. 9 In the clinical setting, despite reports suggesting this association, the mechanism by which metal particles induce fibrosis and stimulate mutagenesis in periprosthetic tissue has not yet been confirmed. In 2015, Sarhadi et al. found genetic abnormalities in periprosthetic tissue, including alterations in genes related to the development of malignant neoplasms. In addition, genetic anomalies were associated with a longer period of contact of the tissue with the implant. 10

In the present report, we present a case of association between high-grade UPS and a THA performed 24 years ago, in a 79-year-old man with bilateral THA. Considering that THA is the gold standard procedure for the treatment of hip osteoarthritis, performed worldwide on a large scale, its association with the development of a malignant lesion is an extremely rare event. However, orthopedic surgeons and radiologists should be alerted and aware of the possibility of an association between alterations in peri-implant tissues caused by debris particles resulting from the degradation of metallic implants and the development of sarcomas in patients with THA loosening. In this regard, early diagnosis and immediate surgical treatment can prevent an unsatisfactory evolution, with the possible emergence of metastases and an unfavorable outcome for the patient.
