# Supplementary material for: High-grade Pleomorphic Sarcoma Associated with Metallosis in a Patient with Total Hip Arthroplasty
Source: Rev Bras Ortop (Sao Paulo). 2021 Oct 13;59(Suppl 1):e17–21. doi: 10.1055/s-0041-1735140 (PMC11254430; doi:10.1055/s-0041-1735140)
Supplement: Related texts

# Research article: Sarcoma pleomórfico de alto grau associado a metalose em paciente com artroplastia total do quadril

**Keywords:** artroplastia de quadril, metais, próteses de quadril, sarcoma

## Abstract

Apesar da relação entre artroplastia de quadril e o desenvolvimento de sarcoma ter sido descrita pela primeira vez na literatura há cerca de quarenta anos, esta associação é extremamente rara. No presente relato de caso, descrevemos a associação entre implante ortopédico e sarcoma de partes moles em um homem de 79 anos submetido a artroplastia primária total do quadril (ATQ) por coxartrose há 24 anos. Foram descritas a evolução clínica e os achados radiográficos e histopatológicos da lesão. No intraoperatório da segunda cirurgia de revisão, foi evidenciada soltura dos componentes acetabular e femoral em associação com extensas áreas de necrose e de metalose. Foi realizado desbridamento da região do quadril e da coxa direita e retirada dos implantes. Devido à extensão da lesão e da necrose, não foi possível realizar nova reconstrução articular. O diagnóstico histopatológico de sarcoma pleomórfico indiferenciado de alto grau associado a extensas áreas de metalose foi estabelecido no tecido adjacente ao implante. O paciente evoluiu com metástases pulmonares e faleceu 6 meses após o diagnóstico. Apesar da raridade da associação, os sarcomas devem ser considerados no diagnóstico diferencial das solturas assépticas, particularmente na presença de metalose no tecido peri-implantar. Pelo nosso conhecimento o período de latência de 24 anos entre a ATQ primária e o estabelecimento do diagnóstico de sarcoma é um dos mais longos relatado até o momento.

## Introdução

A resposta celular às partículas de debris provenientes da degradação de diversos tipos de materiais constitui a base biológica da osteólise peri-implantar que resulta nas falhas das artroplastias totais de quadril (ATQs). 1 Os debris de desgaste dos componentes da prótese ativam o sistema monocítico/macrofágico, promovendo a liberação de citocinas pró-inflamatórias, particularmente de fatores de crescimento, de interleucinas e da proteína ligante do receptor ativador do fator nuclear kappa β (RANKL, na sigla em inglês). A ação integrada destas moléculas de sinalização celular, e em particular a ativação da osteoclastogênese pela interação do RANKL com seu receptor ativador do fator nuclear kappa (RANK), iniciam o processo de osteólise. A ativação do sistema RANK/RANKL resulta no aumento do recrutamento e da atividade dos osteoclastos na interface com o implante, levando à reabsorção óssea e ao afrouxamento e à soltura da prótese. 1

A relação entre artroplastia de quadril e o desenvolvimento de sarcoma foi relatada pela primeira vez em 1984. 2 Em uma revisão compreendendo um período de 30 anos (1974–2003), foi descrita a ocorrência de 46 tumores malignos (41 sarcomas, 4 linfomas e 1 carcinoma epidermóide) relacionados a ATQ. 3

## Relato de Caso

Homem, branco, 79 anos, com coxartrose idiopática bilateral submetido a ATQ primária em 1992 (quadril direito) e 1993 (quadril esquerdo). Nas duas cirurgias, foi utilizado componente acetabular não cimentado composto por liga de titânio revestido em plasma poroso, fixado com parafusos acetabulares, núcleo acetabular de polietileno e haste femoral não cimentada, composta por liga de titânio e superfície rugosa proximal, recoberta por hidroxiapatia e cabeça intercambiável de cromo-cobalto. Em janeiro de 2001, o paciente apresentou soltura do componente acetabular do quadril esquerdo e foi submetido a artroplastia de revisão com troca por prótese acetabular não cimentada. Em setembro do mesmo ano, pelo mesmo diagnóstico, foi submetido a artroplastia de revisão do quadril direito com manutenção da haste e troca do acetábulo por componente cimentado. No intraoperatório dos dois procedimentos cirúrgicos, foram observados desgaste importante do polietileno e focos extensos de metalose no tecido peri-implantar. Em fevereiro de 2016, o paciente apresentou aumento de volume e dor intensa no quadril direito e dificuldade para deambular. Ao exame físico, apresentava aumento do volume da coxa sem flutuação, diminuição do arco de movimento e incapacidade de suportar carga no membro, sem déficit neurológico. Dois meses após o início dos sintomas, foi realizado novo procedimento de revisão no quadril direito, tendo como principal hipótese diagnóstica infecção e como diagnóstico diferencial lesão pseudotumoral por debris de metalose. No intraoperatório, foram evidenciadas soltura dos componentes acetabular e femoral e extensas áreas de necrose tecidual e de metalose envolvendo toda a região periarticular, sem evidência de secreção purulenta ( Fig. 1 ). Foi realizado desbridamento da região do quadril e da coxa direita para retirada dos implantes e coleta de material para cultura microbiológica e avaliação histopatológica. Devido à extensão da necrose tecidual, não foi possível realizar nova reconstrução articular. A avaliação histopatológica confirmou o diagnóstico de sarcoma pleomórfico indiferenciado (SPI) de alto grau e metalose ( Fig. 2 ). Os resultados das culturas microbiológicas intraoperatórias foram negativos. Em julho de 2016, a radiografia do quadril direito evidenciou volumosa massa tumoral em partes moles e extensa destruição do terço proximal do fêmur ( Fig. 3 ). O paciente evoluiu com perda de peso e dispneia, sendo diagnosticado derrame pleural e múltiplos nódulos metastáticos nos pulmões ( Fig. 4 ), e faleceu em agosto de 2016, 24 anos após a realização da ATQ primária.

**Fig. 1:** Cirurgia de revisão do quadril direito em maio de 2016. (A) Radiografia panorâmica da bacia em incidência anteroposterior evidenciando artroplastia total bilateral de quadril. À direita, observa-se sinais de reabsorção proximal do fêmur e migração do componente acetabular para dentro do ilíaco, que apresenta osteólise difusa. Nota-se ainda importante aumento de volume de partes moles na região periprotética. A protése não cimentada à esquerda apresenta afrouxamento do componente acetabular, que se encontra medializado e verticalizado. O componente femoral está fixo. (B) Imagens do intraoperatório mostrando metalose (áreas enegrecidas). (C) Áreas extensas de necrose do tecido adjacente ao implante (setas) que foi desbridado para a retirada dos componentes acetabular e femoral, não sendo possível realizar nova reconstrução articular.

**Fig. 2:** Aspecto histopatológico e perfil imuno-histoquímico da lesão associada ao implante da articulação do quadril direito. (A) Sarcoma pleomórfico indiferenciado de alto grau caracterizado por grau acentuado de anaplasia celular (setas). (B) Debris de desgaste dos componentes da prótese identificado pelos pigmentos enegrecidos (setas) em associação com as células tumorais (*). (C) Ausência de imunomarcação para desmina. (D) Imunopositividade fraca para proteína S-100. (E) Expressão positiva difusa para os anticorpos anti-CD 68 e (F) Anti-Vimentina. (A e B) Coloração hematoxilina e eosina (H&E). Barra de escala: 50µm (A,B), 70µm (C-E) e 100µm (F).

**Fig. 3:** Radiografia do quadril direito realizada em julho de 2016 após procedimento de artroplastia de revisão via acesso posterolateral com remoção dos componentes protéticos. Presença de volumosa massa tumoral em partes moles em associação com extensa destruição do terço proximal do fêmur.

**Fig. 4:** Tomografia computadorizada do tórax. Em julho de 2016, o paciente apresentava derrame pleural e múltiplos nódulos metastáticos em ambos os pulmões e evoluiu para o óbito em agosto de 2016.

## Discussão

As incidências de sarcomas de partes moles na população em geral e em associação com ATQ foram estimadas em entre 0,5 e 2,0 para cada 100.000 indivíduos por ano 4 e em 1,43 para cada 100.000 indivíduos por ano, 3 respectivamente. A associação a implantes mais frequente é com o SPI de alto grau (fibrohistiocitoma maligno, segundo a nomenclatura antiga). Contudo, a associação entre ATQ e outros sarcomas, como osteossarcomas, angiossarcoma e sarcoma sinovial também já foi descrita. 5 6 7 Em um grande estudo populacional, foi demonstrado que os implantes de quadril metal-metal não estão associados a um risco geral aumentado de câncer ou morte por causa oncológica durante um período médio de acompanhamento de 7,4 anos. 8 Porém, neste estudo, não foram incluídos implantes do mesmo tipo utilizado no presente relato (polietileno-metal).

Considerando a data do início das queixas do paciente compatíveis com doença neoplásica (tumoração local, perda de peso e dispneia) com a confirmação histológica do diagnóstico de SPI, o intervalo de tempo entre a ATQ primária e o desenvolvimento da neoplasia foi de 24 anos. Este prazo é consideravelmente superior ao tempo médio de 6,7 anos 3 para o desenvolvimento de sarcomas de partes moles relacionados com implantes ortopédicos.

A carcinogênese induzida por partículas de desgaste e corrosão de metais foi investigada *in vitro* através da exposição de fibroblastos a partículas de cobalto e cromo. As partículas dos metais promoveram a geração de espécies reativas de oxigênio que resultaram em aneuplodia, alterações cromossômicas, fragmentação mitocondrial e danos à rede de microtúbulos do citoesqueleto. 9 No cenário clínico, apesar dos relatos sugerindo esta associação, o mecanismo através do qual as partículas de metais induzem a fibrose e estimulam a mutagênese no tecido periprotético ainda não foram estabelecidos. Em 2015, Sarhadi et al. verificaram anomalias genéticas no tecido periprotético, incluindo alterações em genes relacionados com o desenvolvimento de neoplasias malignas. Além disso, as anomalias genéticas mostraram associação com o maior período de contato do tecido com o implante. 10

No presente relato, apresentamos um caso de associação de SPI de alto grau e ATQ realizada há 24 anos em um homem de 79 anos de idade submetido a ATQ bilateral. Considerando que a ATQ é o procedimento padrão-ouro para o tratamento da osteoartrite do quadril, realizado mundialmente em grande escala, a sua associação com o desenvolvimento de uma lesão maligna é um evento extremamente raro. Contudo, cirurgiões ortopédicos e radiologistas devem ser alertados e estar atentos para a possibilidade da associação entre alterações dos tecidos peri-implantares causadas por partículas de debris provenientes da degradação de implantes metálicos e o desenvolvimento de sarcomas em pacientes com afrouxamento de ATQ. Neste sentido, o diagnóstico precoce e o tratamento cirúrgico imediato podem evitar uma evolução insatisfatória, com o possível surgimento de metástases e um desfecho desfavorável para o paciente.
